# Peripheral and central immune cell reservoirs in tissues from asymptomatic cats chronically infected with feline immunodeficiency virus

**DOI:** 10.1371/journal.pone.0175327

**Published:** 2017-04-06

**Authors:** C. D. Eckstrand, E. E. Sparger, K. A. Pitt, B. G. Murphy

**Affiliations:** 1 Department of Veterinary Microbiology and Pathology, College of Veterinary Medicine, Washington State University, Pullman, Washington, United States of America; 2 Department of Medicine and Epidemiology, School of Veterinary Medicine, University of California Davis, Davis, California, United States of America; 3 Department of Surgical and Radiological Sciences, School of Veterinary Medicine, University of California Davis, Davis, California, United States of America; 4 Department of Pathology, Microbiology and Immunology, School of Veterinary Medicine, University of California Davis, Davis, California, United States of America; CEA, FRANCE

## Abstract

Feline immunodeficiency virus (FIV) infection in cats results in life-long viral persistence and progressive immunopathology. We have previously described a cohort of experimentally infected cats demonstrating a progressive decline of peripheral blood CD4+ T-cell over six years in the face of apparent peripheral viral latency. More recently we reported findings from this same cohort that revealed popliteal lymph node tissue as sites for ongoing viral replication suggesting that tissue reservoirs are important in FIV immunopathogenesis during the late asymptomatic phase of infection. Results reported herein characterize important tissue reservoirs of active viral replication during the late asymptomatic phase by examining biopsied specimens of spleen, mesenteric lymph node (MLN), and intestine from FIV-infected and uninfected control cats. Peripheral blood collected coincident with harvest of tissues demonstrated severe CD4+ T-cell depletion, undetectable plasma viral *gag* RNA and rarely detectable peripheral blood mononuclear cell (PBMC)-associated viral RNA (vRNA) by real-time PCR. However, vRNA was detectable in all three tissue sites from three of four FIV-infected cats despite the absence of detectable vRNA in plasma. A novel *in situ* hybridization assay identified B cell lymphoid follicular domains as microanatomical foci of ongoing FIV replication. Additionally, we demonstrated that CD4+ leukocyte depletion in tissues, and CD4+ and CD21+ leukocytes as important cellular reservoirs of ongoing replication. These findings revealed that tissue reservoirs support foci of ongoing viral replication, in spite of highly restricted viral replication in blood. Lentiviral eradication strategies will need address tissue viral reservoirs.

## Introduction

Feline immunodeficiency virus (FIV) is a lentivirus present in feral and domestic cat populations worldwide. As is true for all lentiviruses, infection is life-long due to irreversible integration of the provirus into genomes of leukocytes including lymphocytes and macrophages, and may be associated with progressive dysfunction of the immune system.[1] Classically, there are three sequential clinical phases of FIV-infection in cats including the acute, asymptomatic, and terminal acquired immunodeficiency phase. In the acute phase there is infection of multiple leukocyte subsets, prolific viral replication and dissemination to many tissue sites including brain, intestinal tract, and primary and secondary lymphoid organs such as the bone marrow, spleen, and lymph nodes.[2–4] The acute stage of infection is followed by an asymptomatic phase lasting months to years where the FIV-infected cat may demonstrate no outward signs of clinical disease despite the presence of a progressive immunopathology.[5,6] Although FIV is capable of infecting multiple types of leukocytes, the hallmark immunopathology of FIV-infected cats is a progressive loss of peripheral blood CD4+ T cells and an inverted CD4:CD8 T cell ratio. Perhaps due to the high costs of keeping experimental animals for protracted time periods, the acute and early asymptomatic phases of infection have received the greatest investigative attention, while less is understood about viral and immunopathogenesis during the late asymptomatic period and the transition into the terminal stage of infection.

Our laboratory has maintained a cohort of four experimentally FIV-infected specific pathogen free (SPF) cats that have been infected for more than six years. At 8–10 months post inoculation, all four FIV-infected cats transitioned into the asymptomatic phase of infection where plasma and peripheral blood mononuclear cell (PBMC)-associated viral *gag* RNA became rare to undetectable.[7] The viral transcription status in the acute and chronic phases of FIV have been intensely documented in these cats.[8–10] Three of the FIV-infected cats have been considered to be progressors, demonstrating the typical immunopathologic hallmark of FIV-infection characterized by very low numbers of peripheral CD4+ T cells. One of the FIV-infected cats has proven atypical in disease progression based on an absolute CD4+ T cell count and CD4/CD8 ratio that remain indistinguishable from two uninfected control cats. This animal has been characterized as a FIV-infected long-term non-progressor (LTNP) cat.[9,11] Additionally, we have demonstrated methylation and deacetylation of histone proteins physically associated with the FIV-promoter in peripheral blood CD4+ T cells isolated during the asymptomatic phase, which is consistent with a condensed chromatin pattern and viral latency.[12] Recently, we demonstrated evidence of active FIV replication in the popliteal lymph nodes in the face of an apparent absence of active viral replication in peripheral blood.[11] Collectively, these findings suggest that viral lymphoid tissue reservoirs are important in the pathogenesis of disease progression and relative to the peripheral blood, lymphoid tissues more accurately reflect viral infection status of the infected cat.[11]

Investigations focused on the acute phase of infection indicate that FIV disseminates to a wide range of tissues including the lymph nodes, spleen, brain, bone marrow, thymus, intestines and other tissues and it is reasonable to believe that these tissues remain tissue reservoirs throughout infection.[3,4,13,14] We hypothesized that leukocyte subsets isolated from spleen, mesenteric lymph node, and intestine of FIV-infected cats during the late asymptomatic phase would demonstrate viral transcriptional activity as demonstrated by the presence of detectable viral RNA through real-time PCR and a novel *in situ* hybridization assay. Additionally, we hypothesized that these tissues would contain replication competent provirus (assessed by *ex vivo* reactivation) and microscopic immunopathologic alterations, as assessed by histology and immunohistochemistry (IHC). These studies are highly relevant to future efforts to develop effective lentiviral suppression and eradiation strategies that require a thorough understanding of cellular and tissue reservoirs of ongoing viral replication.

## Results

### Depletion of circulating CD4+ leukocytes is associated with chronic progressive FIV infection

**Fig 1:**
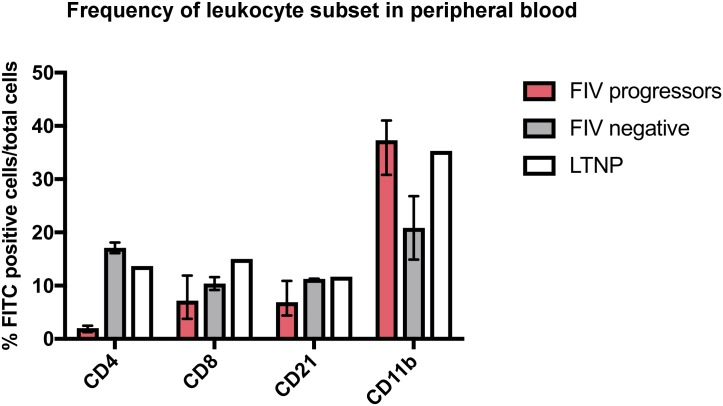
Peripheral blood leukocyte subset frequencies six years post infection. FIV-infected progressor cats show a markedly lower frequency of CD4+ T cells in the peripheral blood compared to uninfected cats. FIV-infected cats (including the LTNP cat) also show a greater frequency of CD11b+ cells. A notable difference in CD8+, and CD21+ and CD11b+ leukocyte subsets was not detected; however statistics were not performed due to low cat numbers. Boxes represent the mean and whiskers represent the numeric range.

Frequencies and absolute number of peripheral blood CD4+ and CD8+ leukocytes, CD21+ B cells, and CD11b+ leukocytes were determined for FIV-infected progressor and long-term non-progressor cats by flow cytometry and compared to uninfected cats (Fig 1). The mean frequency and absolute number of circulating CD4+ T-cells of FIV-infected progressor cats (2.0% and 185 cells/μl ± 93.7, respectively) were greatly reduced relative to uninfected cats at the time of surgery (17.1% and 3770 cells/μl ± 354). The FIV-infected LTNP cat demonstrated a peripheral blood CD4+ leukocyte frequency and absolute count essentially indistinguishable from those of the uninfected cats (13.7% and 3700 cells/μl). Severe peripheral blood CD4+ depletion, as observed for the FIV-infected progressor cats, is a hallmark of FIV infection in cats.[9,11] Due to low animal numbers, statistical comparisons could not be performed. Subjectively, differences in the frequencies of CD8+ and CD21+ leukocytes were not observed in the peripheral blood; however all FIV-infected cats (including the LTNP cat) showed a higher frequency of peripheral CD11b+ cells, which is part of a heterodimeric leukocyte adhesion protein present on feline monocytes, dendritic cells, and neutrophils.[15,16]

### Enriched leukocyte subsets isolated from blood and tissues differed by viral RNA content

**Fig 2:**
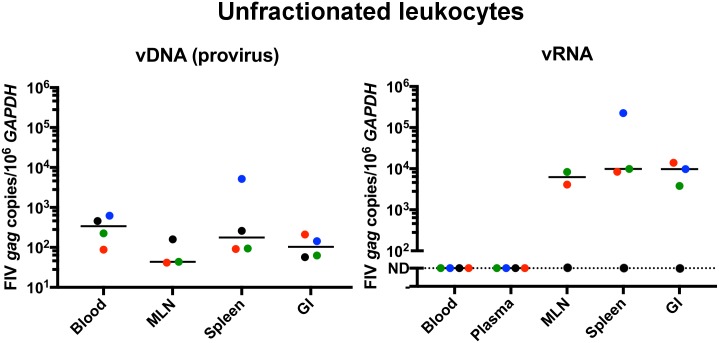
Real-time PCR detection and quantification of proviral and viral *gag* RNA in unfractionated leukocytes from blood and tissue compartments. vDNA was detected in all leukocyte samples from all compartments in all four FIV-infected cats. Note: the MLN was not collected from FIV-infected cat 165 due to the small size of the nodes and surgical safety concerns for this cat. When interrogating leukocytes for the presence of vRNA by real time PCR, plasma and PBMC samples from all cats demonstrated copy numbers below the limit of detection (represented as ND for not detectable). In contrast, abundant vRNA was detected in MLN, spleen, and intestinal tissue compartments from FIV-infected cats, except for cat 184 (black dots). GAPDH was detected in all DNA and RNA samples from all compartments from all cats (including cat 184). The line represents the median. Blue = cat 165, black = cat 184, green = cat 186, red = cat 187 (LTNP cat).

Viral infection (presence or absence of provirus) and transcriptional status (presence or absence of viral *gag* RNA) was determined by real-time PCR in both unfractionated and individually enriched leukocyte subsets (CD4+, CD8+, and CD21+) from peripheral blood, MLN, and spleen. Only unfractionated leukocytes were interrogated from the distal small intestinal tract due to a low number of total leukocytes collected from this compartment. Values for viral nucleic acid load determined for unfractionated leukocytes from each compartment represented the overall FIV replication status for that tissue compartment (Fig 2). Cell-free plasma from each cat was also examined for the presence of viral *gag* RNA (vRNA). Viral *gag* RNA was not detected in the cell free plasma or unfractionated PBMC samples of any of the FIV-infected or uninfected cats, consistent with our previous reports describing an inactive viral transcriptional signature in the peripheral blood of these asymptomatic FIV-infected cats.[7,8] FIV provirus (viral *gag* DNA) was detected in unfractionated PBMC, MLN, spleen and intestinal samples from all infected cats. Importantly, only lymphoid tissue-derived unfractionated leukocytes revealed detectable vRNA, whereas vRNA remained undetectable in unfractionated PBMCs. For an unknown reason, vRNA was not detected from any compartment from FIV-infected cat 184, while control (*GAPDH* RNA) was detected from all of this cat’s tissues. Neither viral *gag* DNA (vDNA) nor vRNA were found in any blood or tissue derived-unfractionated leukocytes collected from the uninfected control cats. These findings support our hypothesis that leukocyte subsets isolated from spleen, MLN, and intestine from FIV-infected cats during the late asymptomatic phase will generally demonstrate ongoing viral replication based on the presence of detectable vRNA by real-time PCR. There were no statistically significant differences in median vDNA or vRNA loads for all FIV-infected cats between compartments when compared by student’s t-test. As previously reported^9^, the LTNP cat 187 showed the lowest vDNA load among all the FIV-infected cats in unfractionated PBMCs and lymph node tissue-derived leukocytes, and also in splenic leukocytes in this study. Interestingly, the intestine-derived leukocytes from this LTNP cat showed a greater mean proviral load (2.1 x 10^2^ vDNA copies/10^6^ GAPDH ± 2.2 x 10^1^) than the progressor cats (9.5 x 10^1^ vDNA copies/10^6^ GAPDH ± 4.9 x 10^1^), and held statistical significance (p = 0.003). In contrast, vRNA load was lower in all tissues for this cat, again with the exception of intestinal-derived leukocytes, where mean viral RNA load was greatest (3.4 x 10^4^ copies vRNA/10^6^ GAPDH ± 1.3 x 10^3^; p = 0.04) among the FIV-infected progressor cats (7.7 x 10^3^ copies vRNA/10^6^ GAPDH ± 3.6 x 10^3^).

**Fig 3:**
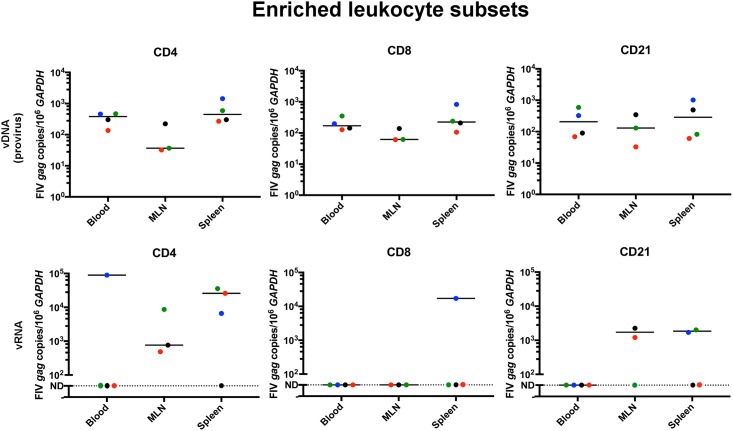
Real-time PCR detection and quantitation of vRNA in CD4+, CD8+ and CD21+ leukocyte subsets enriched from blood and tissue compartments. Provirus was detected in all leukocyte subsets from blood, MLN and splenic compartments. Viral RNA was undetectable (represented as ND for not detectable) in blood PBMC-derived CD4+, CD8+ and CD21+ leukocyte subsets harvested from all FIV-infected cats, except from CD4+ cells from cat 165. CD4+ leukocytes derived from MLN and spleen consistently contained detectable vRNA, while it was intermittently detectable in tissue derived CD21+ leukocytes and was rarely detectable in tissue derived CD8+ T cells. Bars represent the median. Student’s t-tests comparing the level of provirus or viral *gag* RNA between anatomical compartments among leukocytes subsets revealed no statistically significant differences between any tissues. All leukocytes subsets from all compartments in all cats revealed readily detectable *GAPDH* in both RNA and DNA fractions. Blue = cat 165, black = cat 184, green = cat 186, red = cat 187 (LTNP cat).

Proviral DNA was detected in enriched CD4+, CD8+, and CD21+ leukocytes from all examined compartments (PBMCs, MLN and spleen, Fig 3). This finding is consistent with previous reports confirming that these leukocyte subsets are susceptible to FIV infection.[11,17,18] Results were variable when blood and tissue-derived enriched leukocyte subsets were interrogated for the presence of vRNA (Fig 3). Viral RNA was detected in CD4+ cells derived from the blood of one animal (cat 165), representing a rarely detectable vRNA blip in the peripheral blood, as previously described.[11] Viral RNA was detected in MLN CD4+ leukocytes from three out of three cats and in splenic CD4+ leukocytes from three out of four FIV-infected cats. Viral RNA was not detected in PBMC-derived CD21+ or CD8+ leukocytes harvested from any of the infected cats, but was detected in MLN and spleen-derived CD21+ leukocytes in 2 out of 3 (cats 184 and 187) and 2 out of 4 cats (cats 165 and 186), respectively. For CD8+ T cells, vRNA was detected in spleen-derived leukocytes from only one infected cat (165). These results support the concept of active viral transcription in central lymphoid tissues, in spite of rare to undetectable viral replication in the peripheral blood. These results also suggest that tissue-associated CD4+ and CD21+ leukocytes support greater active virus replication in the late asymptomatic phase than CD8+ T cells. Viral *gag* RNA was not detected in any of the enriched leukocytes obtained from the uninfected cats. No statistically significant differences in virus loads were identified between leukocyte subsets or between tissue sites due to the wide variation in viral loads across different cats and different cell subsets.

### *Ex vivo* viral reactivation recovered replication-competent virus from multiple tissue-derived leukocyte subsets

**Fig 4:**
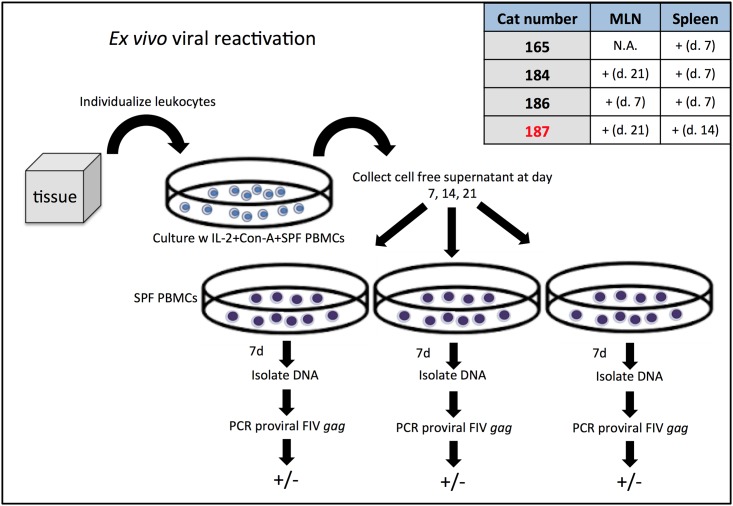
Detection of virus infection in tissues by *ex vivo* viral reactivation. The protocol used for detection of leukocytes harboring replication competent proviruses is shown in this diagram. Replication competent and infectious virus was successfully propagated by *ex vivo* viral reactivation and detected by real-time PCR from all MLN and spleen tissue samples collected from FIV-infected cats as shown in the associated table. A positive detection is indicated by the + symbol, followed by the number of days in culture required for detection of infectious virus. A MLN was not collected from cat 165, and therefore was not tested for this cat (represented by the abbreviation N.A.). The LTNP cat 187 is designated in red and took a longer time to reactivate replication competent provirus from MLN and spleen-derived leukocytes.

Mesenteric lymph node and spleen-derived leukocytes cultured *ex vivo* from all FIV-infected cats demonstrated the capacity to produce infectious, replication competent virus as determined by an *ex vivo* reactivation assay (Fig 4). Clarified cell-free culture supernatants collected from unfractionated tissue-derived leukocytes cultures were passaged onto SPF FIV-negative feline PMBCs on days 7, 14, and 21. After 7 days of secondary culture, real-time PCR was performed to identify the presence or absence of vDNA in SPF PBMC genomic DNA (Fig 4). A positive PCR result indicated that the clarified supernatant contained infectious virus. Infectious virus was detected in culture supernatant at day 7 from splenic leukocytes in all cats, except for the FIV-infected LTNP animal, for which virus reactivation required 14 days in culture. Time in culture required for detectable virus production was generally longer for the MLN samples, ranging from seven days for cat 186, and 21 days for cats 184 and the LTNP cat 187. A MLN was not procured from cat 165. FIV provirus was not detected at any time point for MLN or spleen-derived leukocyte cultures from a FIV-uninfected cat (183, negative control). Statistically, a significant correlation did not exist between the unfractionated leukocyte proviral load of each tissue type from each cat and the days to reactivation (MLN r = 0.49, p = 0.67; Spleen r = -035, p = 0.65).

### Lymphoid tissues show alterations in lymphoid architecture associated with chronic FIV infection

The range of tissue weights for surgically removed sections of spleen, mesenteric lymph node and intestine were between 0.78–2.13 g, 0.18–0.75 g, and 1.25–3.22 g, respectively. Grossly, the mesenteric lymph nodes of FIV-progressor cats were subjectively much smaller in size than those collected from uninfected cats, so much so that a MLN could not be safely surgically procured from FIV-progressor cat 165.

**Fig 5:**
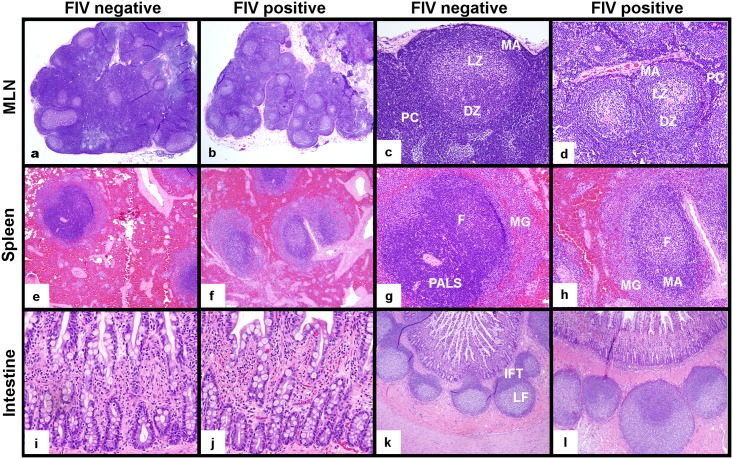
Histologic examination of MLN, spleen and intestine from FIV-infected progressor cats in the late asymptomatic phase and uninfected cats. MLNs from FIV-infected progressor cats were smaller in size and characterized by paracortical atrophy relative to those from uninfected cats (a,b, 20x). MLN follicles from FIV-infected cats revealed active germinal centers with less densely populated dark (DZ) and light zones (LZ), a prominent mantle zone (MA) and a thin, sparsely populated paracortical rim (PC) relative to uninfected cats (c,d, 100x). Spleens of infected progressor cats showed less densely populated white pulp that exhibited indistinct periarteriolar lymphoid sheaths (PALS), prominent active germinal center follicles (F) with expanded mantle (MA) and marginal zones (MG), relative to uninfected cats (e,f 40x, g,h 100x). Notable differences in intestinal mucosal architecture and leukocyte density were not observed between uninfected and FIV-infected cats (200x, i,j). Intestinal Peyer’s patches (lymphoid follicles) of uninfected and infected cats both contained active germinal centers; however, interfollicular T-cell zones (IFT) were better demarcated in intestinal biopsies from uninfected cats relative to infected cats (k,l, 40x).

Histologic examination of the MLNs from the two uninfected control cats revealed multiple active germinal centers with well-demarcated dark, light and mantle zones in the cortical regions of the MLNs (Fig 5a and 5c). The light zone of the germinal centers frequently contained a small amount of extracellular homogeneous/glassy/hyalinized eosinophilic material (presumed immunoglobulin). In both control animals, paracortical regions were densely and thickly populated by small mature lymphocytes. MLNs were analyzed from two FIV-progressor cats (184 and 186) and both demonstrated two striking histologic features (Fig 5b and 5d). The first feature was marked paracortical atrophy characterized by a thin paracortical rim with a scarcity of leukocytes, a decreased distance between the medullary sinuses and capsule, and decreased space between cortical follicles. A second histologic feature of MLNs from FIV-infected progressor cats related to MLN dark and light zones that were less densely populated with leukocytes and showed increased clear space between cells. These findings were consistent with mixed lymphoid hyperplasia and follicular involution.[19] Similar to the uninfected cats, the germinal centers of FIV-progressor cats contained acellular eosinophilic material (presumed to be immunoglobulin) within the germinal center light zone. MLN sampled from the LTNP cat revealed well-populated paracortical zones and follicular domains similar to those of the uninfected cats.

Splenic tissue from the uninfected cats demonstrated quiescent follicular domains with poorly delineated germinal center dark and light zones and well-populated periarteriolar lymphoid sheaths (PALS, Fig 5e and 5g). In contrast, splenic tissues from the FIV-progressor cats (3 cats) revealed large active follicular domains with expanded germinal center light zones, and prominent mantle zones (Fig 5f and 5h). Splenic PALS were not well identified in spleens from FIV-progressor cats on H&E stained sections. The spleen harvested from the LTNP cat showed both active germinal centers in follicular domains and well populated PALS.

Interestingly, intestinal biopsies revealed no overt architectural differences in the mucosa between FIV-infected and uninfected cats on histologic exam (Fig 5i and 5j). The Peyer’s patches examined from biopsies of uninfected cats demonstrated prominent follicular and interfollicular zones (Fig 5k) and one cat had abundant brightly eosinophilic acellular material in the germinal center light zone. Intestinal biopsies from FIV-infected progressor cats exhibited active germinal centers and poorly delineated interfollicular T-cell zones (Fig 5l). Intestinal Peyer’s patches from the LTNP cat demonstrated active follicular germinal centers, and numerous small lymphocytes populating the interfollicular T-cell zone similar to the uninfected cats.

### Distinctive alterations in leukocytes subset frequency and distribution is associated with FIV localization within different lymphoid tissues

**Fig 6:**
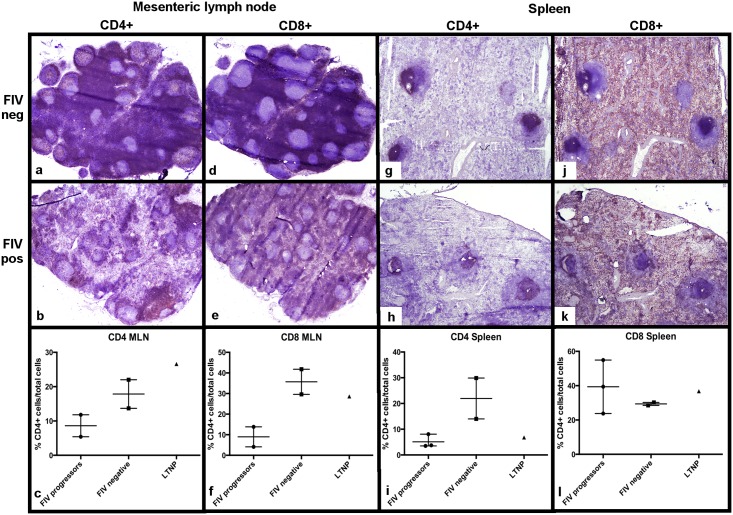
Determination of CD4+ and CD8+ leukocyte frequency in MLN and spleen from FIV-infected and uninfected cats. A severe CD4+ depletion is observed in the paracortex and medullary cords of MLN and in periarteriolar lymphoid sheaths of the spleen of FIV-infected progressor cats relative to uninfected cats (a,b,g,h), consistent with the flow cytometry data (c,i). A notable CD8+ depletion is also evident in the paracortex of MLNs from FIV-infected progressor cats relative to uninfected cats (d,e), and supported by flow cytometric analysis (f). IHC analysis for CD8+ T cell distribution demonstrates diffuse staining of the red pulp in both infected and uninfected cats (j,k) whereas splenic CD8+ T cell frequencies are variable among cats by flow cytometry (l). For flow cytometry data, each point represents one cat, the horizontal bar represents mean, and the whiskers represents the range. The LTNP cat’s flow cytometry value is a single triangle.

Findings from immunohistochemical analysis of MLNs for T leukocyte subset frequency and distribution for the two FIV-progressor cats (184,186) shared similarities that included diffuse depletion of lymphocytes within germinal centers, paracortical zones, and medullary cords. Immunohistochemistry (IHC) assay for CD4+ leukocytes demonstrated greatly reduced numbers and irregular distributions of CD4+ leukocytes throughout the MLNs of both FIV-progressor cats examined relative to uninfected cats. Furthermore, these IHC results corroborated the decreased frequency of CD4+ cells in the MLN determined by flow cytometry (Fig 6a–6c). Similar findings were observed with IHC analysis of MLNs for CD8+ T cell frequency which revealed a patchy and pauci-cellularity in FIV-infected cats relative to uninfected cats. Again, IHC analysis for CD8+ T cell frequency was consistent with the flow cytometry data showing CD8+ T cell depletion in the MLN (Fig 6d–6f). Of note, a subjective increase in the number of CD8+ T cells within the mantle zone of cortical lymphoid follicles was observed for MLNs from FIV-infected cats relative to uninfected control cats (data not shown). The MLN harvested from the LTNP cat revealed paracortical zones and medullary cords that were densely populated with numerous CD4+ and CD8+ cells that were not consistent with lymphoid depletion. These IHC data were again consistent with flow cytometric analysis of T cell subset frequencies in the MLN of the LTNP cat (Fig 6c and 6f).

Findings from IHC analysis for CD4+ and CD8+ leukocyte distribution within the spleen were similar among the three FIV-infected progressor cats (165, 184 and 186) and included a diffuse CD4+ leukocyte depletion of periarteriolar lymphoid sheaths and disorganized lymphoid follicles with scattered embedded CD4+ and CD8+ cells (Fig 6g–6l). The splenic red pulp of both FIV-positive and FIV-negative cats contained large numbers of CD8+ T cells with no identifiable difference between groups, however the flow cytometric data suggested a greater proportion of CD8+ T cells in FIV-infected cats (Fig 6j–6l). Interestingly, IHC analysis of CD4+ leukocytes in the spleen from the LTNP cat demonstrated slight pauci-cellularity of the PALS T-cell regions (data not shown), which was supported by flow cytometric analysis revealing a CD4+ cell frequency similar to values of the progressor cats. Based on IHC analysis, there was no difference identified in the distribution of the splenic CD8+ cells within red pulp between the LTNP cat and FIV-negative cats.

**Fig 7:**
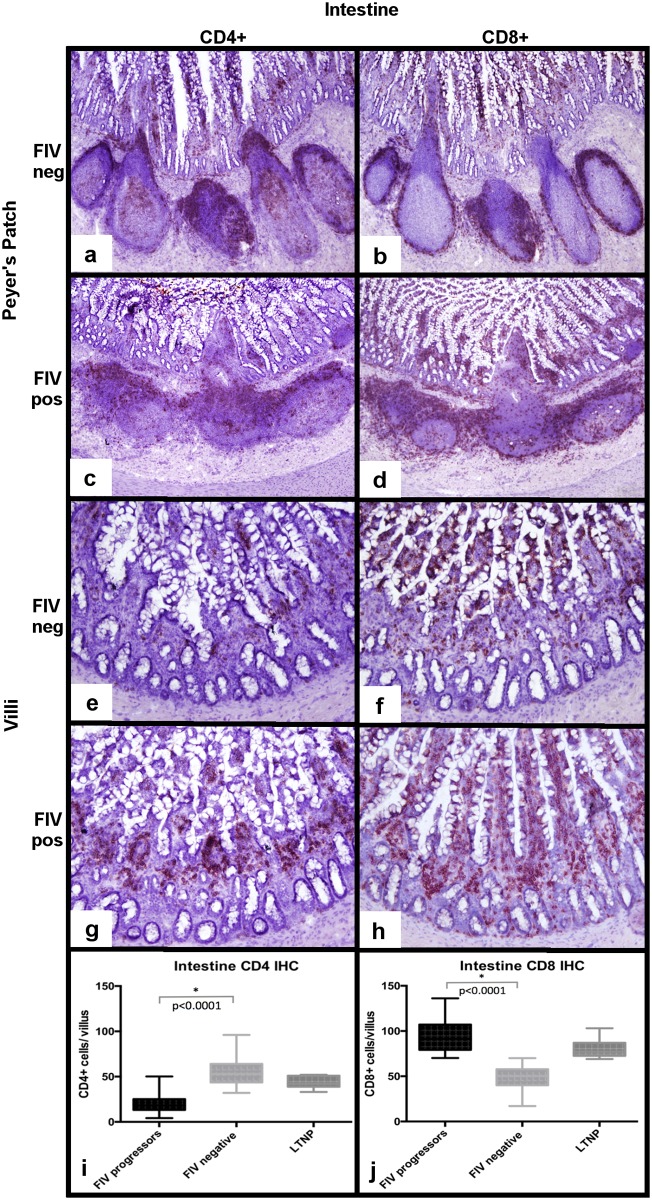
Determination of CD4+ and CD8+ leukocyte frequency within intestinal tissues from FIV-infected cats. Relative to the FIV-uninfected cats (a), FIV-progressor cats show a less discrete germinal center architecture, merging of follicles and patchy, sparsely populated interfollicular T-cell zones (c). T-cell zones of the Peyer’s patches are expanded by CD8+ T-cells in FIV-progressor cats (d), relative to dense discrete CD8+ cell aggregates within tissues of the uninfected cats (b). Intestinal villus lamina propria of biopsies from FIV-progressor cats is nearly completely depleted of CD4+ leukocytes relative to uninfected cats (e,g,i). Interestingly, intestinal tissues from FIV-progressor cats show greater numbers of villus CD8+ T relative to uninfected cats (f,h,j).

Intestinal Peyer’s patches were available for evaluation from the three FIV-progressor cats for IHC, but not from the LTNP due to the absence of a Peyer’s patch in the cryopreserved section of intestinal tissue. For the FIV-progressor cats, IHC analysis for CD4+ leukocyte distribution in Peyer’s patches demonstrated sparsely populated and poorly demarcated interfollicular T-cell zones that occasionally merged with one another relative to the densely populated and discrete T-cell zones from uninfected cats (Fig 7a and 7c). Peyer’s patches from FIV-infected cats demonstrated a subjective increase in the number of CD8+ T cells in germinal centers and interfollicular regions and a distribution pattern similar to that of CD4+ cells (unevenly distributed) (Fig 7b and 7d). When examining the intestinal mucosa of uninfected cats, CD4+ leukocytes were distinctly present in the middle third of villi, and formed dense clumps, the density of which tapered towards the villus tips (Fig 7e). There was a notable and statistically significant reduction of CD4+ leukocytes in the villous lamina propria of intestinal mucosal tissues from FIV progressor cats relative to the uninfected cats, consistent with CD4+ cell depletion (Fig 7g and 7i). The distribution of CD8+ T cells in Peyer’s patches from uninfected cats was characterized by scattered small aggregates of positive cells evenly distributed throughout the length of the villi (Fig 7f). A distinction was not made between lamina proprial and intraepithelial dwelling CD8+ T cells. Interestingly, intestinal tissues from FIV-progressor cats revealed a marked and statistically significant increase in the number of CD8+ T cells throughout the villi compared to uninfected cats (Fig 7h and 7j). Intestinal biopsies from the LTNP cat demonstrated villus CD4+ leukocyte counts indistinguishable from uninfected cats, and a slight, but statistically significant increase in villus CD8+ T cells, similar to the FIV-progressor cats (Fig 7i and 7j).

**Fig 8:**
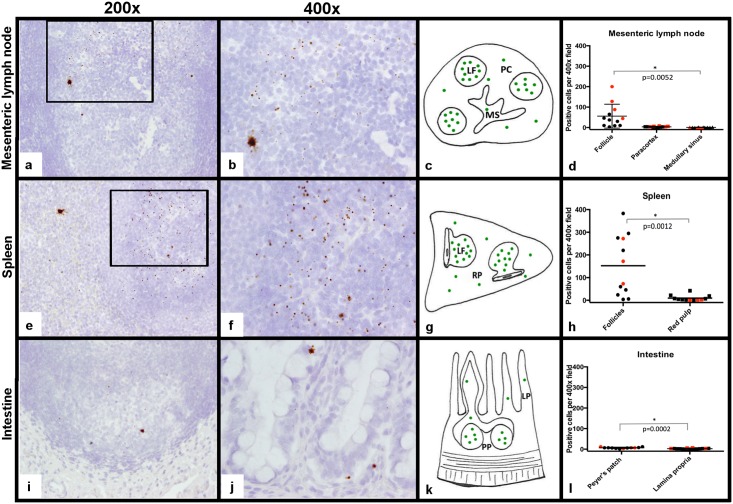
Detection of FIV RNA in the MLN, spleen and intestines. FIV RNA localization in tissues was measured by *in situ* hybridization using RNAscope and represented by brown chromogen dots. Viral RNA was concentrated in lymphoid follicles (LF) within the MLN (a, 200x magnification). At higher magnification (400x), the viral RNA signal was predominantly represented as a single dot, but rare cells were distended with brown chromogen that extended into stellate cell processes consistent with dendritic cell morphology (b). FIV RNA was less frequent in the paracortex (PC) and medullary sinus (MS) regions (c, d). Similarly, FIV RNA was concentrated in the lymphoid follicles of the spleen, and was less frequent in the red pulp (RP, e-h). Within intestinal tissue, FIV RNA was again most concentrated in lymphoid follicles of the Peyer’s patches (PP, i-l) and less frequently detected in the lamina propria (LP) of villi (j, 400x). Red dots on the graphs represent the LTNP cat.

By *in situ* hybridization using RNAscope, FIV RNA-positive cells were identified in the follicular, paracortical and medullary sinus zones of MLNs, with the greatest concentration of positive cells in the light zone of cortical lymphoid follicle germinal centers (Fig 8a–8c). *In situ* hybridization analysis of MLNs revealed similar findings shared by the FIV-progressor cats and LTNP cat. These findings specifically revealed that the highest concentration of FIV-RNA positive cells were localized to the germinal centers of the follicular zones and associated with rare scattered positive cells in the paracortex and medullary sinuses. Occasional RNA-positive cells, predominantly present in follicular domains, demonstrated a unique staining pattern in which chromogen filled the cell body and extended into stellate cell processes, suggesting these RNA-positive cells may be dendritic cells (Fig 8a and 8b).

Similar to findings for MLNs, analysis for viral RNA localization within spleen by *in situ* hybridization revealed that FIV RNA-positive cells were most concentrated in the germinal centers of the splenic white pulp and were less frequently detected in the red pulp for all FIV-infected cats (Fig 8e–8h). Stellate cells containing abundant FIV RNA (similar to those observed in the MLNs) were also observed in the spleen. These cells were rare, but present in all FIV-infected cats and were noted in both follicular and red pulp regions.

Analysis of intestinal tissues by *in situ* hybridization again demonstrated viral RNA-positive cells mostly concentrated in germinal centers with less frequent RNA-positive cells present in the lamina propria of the villi (Fig 8i–8l). Interestingly, the number of FIV RNA positive cells per high power field in the intestinal compartment was far fewer than numbers detected within the MLN and the spleen, suggesting that viral production within the intestinal mucosa is reduced relative to the MLN and spleen during the late asymptomatic phase of FIV infection.

## Discussion

In this study we showed that this cohort of FIV-infected cats in the late asymptomatic phase demonstrate active viral replication in multiple central tissue sites with a relatively quiescent viral replication status in the peripheral blood and concurrent CD4+ leukocyte depletion in peripheral blood and tissue sites. We used a novel *in situ* hybridization assay based on RNAscope technology to identify and localize viral RNA in tissue, revealing specific microanatomical reservoirs of active viral replication. An additional advantage of this *in situ* hybridization assay is the ability to concurrently detect viral RNA from multiple genomic loci, thus increasing the assay's sensitivity for low levels of viral transcription. This sensitivity was exemplified by the fact that viral RNA was detectable in tissues by *in situ* hybridization in all four of the FIV-infected cats, while real-time PCR failed to detect viral *gag* RNA in tissue-derived leukocytes of one FIV-progressor cat (184). Beebe et al. investigated the distribution of viral nuclei acids in the acute stage of FIV infection and showed by traditional *in situ* hybridization methods that lymphoid tissues including the intestine harbor viral RNA.[2] However, to our knowledge, this is the first report utilizing *in situ* hybridization for tissue localization of FIV-RNA in the late asymptomatic phase of FIV-infection in cats.

In these chronically infected asymptomatic cats, viral RNA was most concentrated within the lymphoid follicles of all of the tissues examined (mesenteric lymph node, spleen, and distal small intestine), as determined by *in situ* hybridization. Previous investigations have demonstrated virus in lymphoid follicles during the acute stage of FIV infection, which together with the results presented here lends support to the hypothesis that lymphoid follicles are long-term microanatomical reservoirs of viral persistence.[2,13] Investigations into determining the immunologic factors that support viral replication in lymphoid follicles are warranted, however it seems plausible that FIV replicates effectively in lymphoid follicles because of the immunologically active state of germinal centers and FIV’s preference to infect and replicates within activated lymphocytes.[20–23] Also interesting are studies that elucidate the role of OX-40 (CD134) in germinal center biology, which also happens to be the primary receptor utilized by FIV for cellular entry.[20,24] In mice it has been shown that the ligand for OX-40 is expressed on lymphoid tissue dendritic cells and leads to an accumulation and possible retention of T cells within lymphoid follicles.[25] Additionally OX-40 has been demonstrated on murine activated B cells (as well as feline CD45R+ B cells) and has an important role in B cell help and antibody switching.[26–28] Connections, if any, between the activated microenvironment of the germinal center, expression of OX-40, and lymphoid tissue FIV replication have yet to be elucidated. Previous investigations in SIV-infected macaques have similarly demonstrated the presence of SIV-RNA in lymphoid follicles by *in situ* hybridization. Additional reports suggest that the microenvironment of the lymphoid follicle is such that CD8+ cytotoxic T-cells are excluded from lymphoid follicles of rhesus monkeys, thus creating an immunoprivileged site that provides a haven for viral replication.[29] In the study described here, we identified the presence CD8+ T cells within lymphoid follicles, thus making it less likely that lymphoid follicles are immunoprivileged sites for FIV replication because of an absence of cytotoxic T-cells.

Normal lymphoid follicles are heavily populated by B-cells with fewer scattered follicular dendritic cells, CD4+ T cells, and even fewer CD8+ T cells. It remains undetermined which of these follicle-associated leukocyte subsets contribute to the production of FIV RNA, but this could be elucidated by performing a dual staining assay using both *in situ* hybridization and immunohistochemical procedures to immuophenotype the FIV RNA positive cells. We did however perform immunomagnetic separation of CD4+, CD8+, and CD21+ cells from the MLN and spleen which demonstrated viral *gag* RNA (real-time PCR) consistently within CD4+ and CD21+ leukocytes, and rare to undetectable viral *gag* RNA in CD8+ T cells. Our group has previously reported two leukocyte subsets (CD4+ and CD21+) that contribute to the production of FIV *gag* RNA enriched from popliteal lymph nodes of chronically infected cats.[11] In the study reported here, we expanded our investigation to include CD8+ T cells as a possible chronic cellular reservoir, as these leukocytes have previously been shown to be susceptible and permissive to FIV infection.[17,30] Our results demonstrated that in the late asymptomatic phase, both tissue and blood derived-CD8+ T cells were infected (i.e. contain provirus,) but viral *gag* RNA was only rarely detectable in tissue-derived CD8+ T cells and was undetectable in blood-derived CD8+ T cells.

One limitation of our study is the fact that histiocytic cells (monocytes, macrophages, dendritic cells) were not investigated as a potential cellular reservoir, but are of known importance in FIV infection.[2,13,31] This was primarily due to the difficulty in positively (specifically) selecting/enriching viable tissue histiocytes with the antibodies currently available. Interestingly, *in situ* hybridization revealed the presence of a rarely present viral RNA-containing cell type in tissue with stellate-like processes radiating outwards. Given their apparent cytomorphology, these cells are most likely dendritic cells. However we did not further interrogate the cell type through ancillary techniques. In the acute stage of FIV it was demonstrated by Rogers et al. that dendritic cells in lymphoid follicles contain FIV RNA. These findings highlight the importance of improving techniques for isolating histiocytic cells for future investigations into FIV reservoirs.

In addition to investigating the transcriptional activity (presence of viral RNA) of FIV in tissue leukocytes, we demonstrated replication competency (ability of infected cells to produce infectious virus) of both MLN and spleen-derived leukocytes. This was assessed by an *ex vivo* viral reactivation assay, which is considered the gold standard for assessing replication competency and productivity. The reactivation assay, which has also been referred to as a viral outgrowth assay, was described by Siliciano and Siliciano, and can be modified to quantify the size of the reservoir by creating serial dilutions of leukocytes.[32] One limitation to this assay is that even though stimulatory compounds (such as concanavalin-A) are added to induce transcription and proliferation, non-inducible proviruses, or those resistant to induction, exist and are resistant to stimulation. Non-inducible proviruses will be missed by this assay thus yielding a false negative or reduced quantitative result. In this study, infectious virus was successfully propagated from the MLN and spleen-derived leukocytes of all FIV-infected cats in which tissues were obtained. The number of days to produce infectious virions in the supernatant varied, with spleen leukocytes generally producing virus sooner (within 7 days) of culture, while MLN leukocytes took longer (between 7–21 days). This result is also in concurrence with the *in situ* hybridization results, which showed that a greater number of cells are transcriptionally active in the spleen than the MLN. But calculations did not indicate a significant correlation between proviral load and time to reactivation. Tissues derived from the FIV-LTNP cat took longer to reactivate than the FIV-progressor cats (14 and 21 days for spleen and MLN respectively), which is similar to what we have previously reported for this LTNP cat and may due to a lower proviral load.[11]

Previous reports investigating alterations in leukocyte subsets associated with FIV infection have primarily focused on the peripheral blood compartment, while few have investigated alterations in tissues. Lymph node tissue has been examined most frequently, and reports consistently indicate nodal CD4+ depletion occurs as the disease progresses.[11,33,34] This study expanded on previous reports by reporting morphologic and immunohistochemical changes in spleen and intestines as well as lymph node tissue of the FIV-progressor cats. Interestingly, some of the differences identified through immunohistochemistry were not overtly evident in hematoxylin and eosin (H&E) stained sections. For instance, H&E stained MLN and splenic tissue demonstrated leukocyte depletion in the paracortical and periarteriolar lymphoid sheaths zones, respectively, while there was no appreciable leukocyte depletion in the H&E stained sections of intestine. Immunohistochemistry assays for CD4+ antigen on the MLN and spleen confirmed CD4+ cell depletion in the aforementioned areas, but also revealed severe depletion of CD4+ leukocytes in the mucosa of the intestinal villi, a feature not appreciated in H&E-stained sections. Another interesting finding of the study was a marked increase in the number of intestinal villus-associated CD8+ cells, which likely is the reason leukocyte depletion was not appreciated by H&E stained sections. This expansion in intestinal CD8+ leukocytes is similar to what has been reported in chronically SIV-infected macaques and HIV-infected humans (although rectal tissue was used in the mentioned HIV study.[35,36] An investigation by Howard et, al., in chronically FIV infected cats (10–16 months PI) also demonstrated a depletion of CD8+ leukocytes, but focused specifically on intraepithelial CD8α+ leukocytes. Here, we did not differentiate between intraepithelial and lamina proprial CD8+ cells or different CD8+ subsets.

Lymphoid follicles of all of the FIV-infected cats (including the LTNP) of all tissues had demonstrated active germinal centers with generally poorly demarcated light and dark zones, relative to control animals who’s germinal center regions (if present) were well demarcated. Again, immunohistochemistry further defined the disorganization of follicular zones in FIV-progressor cats relative to uninfected cats. It also demonstrated a subjective increase in the numbers of CD4+ and CD8+ cells scattered throughout the follicles similar to what we have previously reported for lymph node lymphoid follicles.[11]

We have previously characterized one of our FIV-infected cats as a long-term non-progressor (LTNP, cat 187) based upon the fact that this animal persistently maintains a peripheral blood CD4+ T cell count indistinguishable from the uninfected cats, has a reduced peripheral blood and lymph node tissue proviral and viral *gag* RNA load, and requires a longer time to reactivate viral production from cells culture *ex vivo*.[11] In this report we have further characterized this LTNP cat. In this study the LTNP cat had a lower proviral load than the FIV-progressor cats in unfractionated leukocytes derived from all of the examined tissue compartments, except the intestinal compartment. Additionally, this cat had a lower proviral load in all three examined leukocyte subsets (CD4+, CD8+, and CD21+ cells), though not of statistical significance. With regards to viral *gag* RNA, the LTNP cat again had reduced viral *gag* RNA loads in all compartments compared to the FIV-progressor cats, except for in the intestinal tract. We do not understand the reason why the LTNP cat had greater intestinal proviral DNA and FIV *gag* RNA loads than the FIV-infected progressor cats, but this finding was also supported by the *in situ* hybridization assay, which demonstrated an abundance of FIV RNA in the intestinal Peyer’s patches relative to the progressor animals. One could hypothesize that this LTNP cat is entering a stage of viral activation that the progressor cats already encountered. Another possibility to explain why this LTNP cat had higher proviral and vRNA load in the intestine is due to the fact that this cat had significantly greater absolute numbers of the leukocyte subsets that FIV targets, relative to the FIV-infected progressor cats. Viral load in this study was normalized to the cellular *GAPDH* gene and thus the results may not fully account for drastic differences in absolute leukocyte count when comparing the progressor cats with massively depleted CD4 T cells (for example) to the LTNP cat who has a relatively normal CD4 T cell count. This represents a limitation of our study, and interpretation of these data would have been more accurately determined by normalizing to leukocyte count.

There are a number of other shortcomings in this investigation that should be discussed. For one, low numbers of animals hindered our ability to apply statistics to some of the data. Additionally, given the goal of survival surgical biopsies, there was a failure to collect complete tissue sets from all animals (one MLN could not be safely removed from one of the FIV-progressor cats). Lastly, while we were able to demonstrate active viral transcription and replication competency, we were not able to detect viral protein in tissues even though attempts were made using both Western blot and immunohistochemical techniques. This likely reflects the relative insensitivity of antibody-based virologic techniques.

In conclusion, we have demonstrated that there is active viral transcription in lymphoid tissue sites (mesenteric lymph node, spleen, and intestinal tissue) in the late asymptomatic phase of FIV infection, which contrasts with the quiescent transcriptional status in the peripheral blood. Ongoing tissue-based viral transcription/replication may play a role in the progressive depletion of peripheral CD4+ and CD8+ leukocytes. Additionally, we employed a novel *in situ* hybridization that increased our ability to detect viral RNA and also facilitated the localization of viral replication to tissue associated lymphoid follicles. Precisely defining the phenotype of the cells responsible for viral transcription will require further studies. These tissues and microanatomical foci of ongoing replication should be considered persistent viral reservoirs and future eradication strategies must take into account their importance in the pathogenesis of this disease.

## Materials and methods

### Animals, peripheral blood and abdominal tissue procurement

Six specific pathogen free (SPF) kittens were purchased from the breeding colony of the Feline Nutrition and Pet Care Center, University of California, Davis (UC Davis). At the time of purchase, kittens ranged in age from 4 to 5 months and were housed in the Feline Research Laboratory of the Center for Companion Animal Health, UC Davis. Four kittens were intramuscularly inoculated with FIV-C subtype (Paddy-Gammer strain) viral inoculums [37] (kittens 165, 184, 186 and 187) and two control kittens (183 and 185) were mock-inoculated intramuscularly with 1 ml of sterile culture media and monitored as previously described [7]. The FIV-C biological isolate was provided by Drs. E. Hoover (Colorado State University) and N. Pedersen (UC Davis). The study cats were housed in cat groups (4 cats FIV positive and 2 cats FIV negative) and maintained in individual rooms that were cleaned daily. The rooms had multiple perches and a variety of cat toys and boxes for enrichment, play and exercise. The cats were fed, observed and litter boxes changed on a daily basis. Room lighting cycles were seasonally adjusted. FIV infected cats and two uninfected age-matched uninfected control SPF cats have been maintained in group housing under SPF conditions for monitoring of virus load, alterations of CD4+ T cell counts and clinical disease consistent with an acquired immunodeficiency according to an animal protocol approved by the University of California Davis Institutional Animal Care and Use Committee (IACUC; protocol #18155). For the current study, the four experimentally FIV-C infected and two uninfected control cats in their sixth year post-inoculation (between 316–333 weeks post-inoculation, wpi) underwent physical examination, collection of 10 mls of peripheral blood, and non-terminal abdominal surgery under general anesthesia to harvest representative sections of spleen, mesenteric lymph node, and distal small intestine (SI, resection and anastomosis) at the jejunal/ileal junction. An approximately 2 cm long section of the distal splenic tail was resected by double hemostat isolation and scalpel blade transection and immediately immersed in sterile ice-cold hank’s balanced buffers solution (HBSS). Hemostasis required combinatorial horizontal mattress suture closure and SURGICEL^®^ hemostat (Ethicon). One to two partial to complete mesenteric lymph nodes were isolated from the small intestinal mesentery by blunt dissection of all cats except FIV-infected cat 165, whose mesenteric lymph nodes were considered too small to safely biopsy (potential for vascular injury). An approximately three cm long intestinal section at the jejunal/ileal junction was collected from each cat by a resection and anastomosis technique using Doyen forceps, scalpel blade transection, and suture reapposition. Surgical procedures, anesthesia and post-operative pain management protocols were also IACUC-approved and employed combinatorial injectable, inhalational, oral and transmucosal pharmaceuticals. The pre-, surgical and post-surgical pharmaceutical protocols used in these cats were identical to those previously described with the addition of post-surgical oral antibiotics (clavulanic acid, Clavamox, at 6.25mg/lb) for a total of 14 days.[11] Post-surgery, the cats were housed individually to allow for personalized, twice daily monitoring of attitude, pain, appetite, body temperature, surgical incision healing, hydration, and bowel movements. Cats were offered food after full recovery from anesthesia (same day as surgery). Cats returned to their group housing arrangement within 6 days post-surgery.

### Peripheral blood and tissue leukocyte collection and flow cytometry

PBMCs were harvested from whole blood by Ficoll-Hypaque (Sigma, St. Louis, Mo.) density gradient centrifugation. Splenic, MLN and SI tissue samples were weighed after the aseptic removal of associated adipose tissue and intestinal contents. For each cat, sections of lymph node, spleen and SI were fixed in 10% buffered formalin and cryopreserved for future histologic and immunohistochemical studies. MLN and splenic tissues were also aseptically dissociated by manual disruption and processed for leukocyte preparations by filtration through a 70 um mesh sieve (BD Falcon). Small intestine-associated leukocytes were isolated by a combination of mechanical and enzymatic digestion techniques. Intraepithelial lymphocytes were collected as described by Howard et al. with the modification that 3 cm of intestine was used and 5 mls of spin media.[38] Lamina proprial lymphocytes were collected by putting the remaining intestinal pieces in 5 mls of spin media plus 15mg of dispase II enzyme (Roche), incubated at 37°C for 10 minutes and mechanically dissociated using the gentleMACS Dissociator (Miltenyi Biotec). The supernatant was strained through a #40 mesh filter (BD Falcon) and centrifuged at 400g for 10 minutes at 16°C. The supernatant was discarded and the lymphocytes were collected from the cell pellet by Percoll gradient centrifugation as described.[38] Isolated PBMC and tissue-derived leukocytes were resuspended and enumerated using both an automated cell counter (Coulter ACT diff, Beckman Coulter) and a manual hemocytometer.

Unfractionated leukocytes prepared from MLN, spleen and blood were assayed for cellular subset frequencies by flow cytometry. Too few leukocytes were collected from the SI sections to perform flow cytometry. Antibodies used for flow cytometry included anti-feline CD4 (clone FE1.7B12), anti-feline CD8 (clone FE1.10E9), anti-canine CD21 (clone CA2.1D6), anti-canine 11b (clone CA16.3E10), and anti-feline MHC II (clone 42.3). All of the antibodies were obtained from the laboratory of Dr. Peter Moore (UC Davis). Frequency of total cells expressing a specific marker was determined by flow cytometry, using indirect immunofluorescence with a secondary fluorescein isothiocyanate (FITC)-conjugated horse anti-mouse IgG (Vector Laboratories Inc.) as previously described.[7] Frequencies were determined using a Cytomics FC500 flow cytometer (Beckman Coulter) and FlowJo v8.6.3 software (Treestar) over 110,000 total events.

### Peripheral blood and tissue leukocyte subset enrichment and real time PCR assays for viral infection

CD4+, CD8+ and CD21+ subsets were isolated from freshly isolated PBMCs, MLN, and splenic leukocytes (between 1.75 x 105–1 x 10^7^ total cells by positive immunomagnetic selection using primary antibodies described above, MS immunomagnetic columns (MACS Separation Columns, Miltenyi Biotec Inc.) and goat-anti mouse IgG-microbeads (Miltenyi Biotec Inc.) as previously described.[7,11] Leukocyte subsets were determined to be >95% pure by flow cytometry and subsequently interrogated for the presence of cell-associated viral *gag* DNA (provirus) and viral *gag* RNA (vRNA).

Viral RNA was isolated from clarified feline plasma and cell-associated RNA and DNA were co-isolated using commercially available kits (QIAmp Viral RNA Minikit and AllPrep DNA/RNA Mini Kit, Qiagen) according to manufacturer's instructions. Plasma vRNA and cell-associated RNA were DNase treated (Turbo DNase, Ambion) and reverse transcribed using the First-Strand cDNA Synthesis System for Quantitative RT-PCR (OriGene). Real-time PCR was performed in triplicate on an Applied Biosystems 7300 Real-Time PCR System with FIV_QT gag_ primers under conditions previously described.[7] Importantly, a negative control reaction excluding reverse transcriptase was included for each set of cDNA samples to ensure RNA samples were not contaminated with DNA. This real-time PCR assay has a detection limit of approximately 10 copies of FIV *gag* cDNA (data not shown) in cellular samples or 10^3^ copies of FIV *gag* cDNA per ml of feline plasma. Quantification of plasma vRNA copy number was based on a standard curve previously described.[39] For assay of cell-associated vDNA and vRNA, real-time PCR assays of feline *GAPDH* were included in parallel using the same nucleic acid samples to normalize nucleic acid concentration as previously described.[11]

### *Ex vivo* viral reactivation

To determine if the tissue-derived leukocytes contained replication-competent virus, an *ex vivo* reactivation assay was performed as previously described for PBMCs isolated from FIV-infected cats with the following modifications.[11,12] Approximately 5 x 10^6^ MLN or splenic-derived leukocytes were cultured *ex vivo* with allogeneic SPF PBMCs (2.5 x 10^6^) in RPMI media (Hyclone) supplemented with 100 units/ml human IL-2 (NIH AIDS Reagent Program) and 5μg/ml of mitogen concanavalin-A (Con-A; ThermoFisher Scientific). Due to reduced numbers of total leukocytes collected from cat 184 (from both MLN and spleen) only 3 and 4.25 x 10^6^ leukocytes were used in the reactivation assay for MLN and spleen respectively. Cultures were passaged as previously described and clarified cell-free supernatants were collected on days 7, 14, and 21 for cryopreservation.[11] At a later time, collected supernatants were independently passaged onto approximately 7 x 10^6^ Con-A activated allogeneic SPF PBMC. Cultured allogeneic cells were subsequently assayed for the presence of *gag* DNA (provirus) by real-time PCR seven days after inoculation with clarified supernatant. Detectable vDNA was interpreted to be consistent with successful reactivation from tissue leukocytes of infected cats. Fresh MLN and spleen leukocytes from all four FIV positive cats, and one negative control cat (185) were assayed.

### Immunohistochemistry and image analysis

Sections of MLN, spleen and intestines were collected for histological examination to assess microscopic morphologic differences between FIV-infected and uninfected cats. Immunohistochemistry (IHC) was performed on seven-micron thick, unfixed, snap-frozen MLN, spleen and SI sections mounted on positively charged slides. Sections were fixed in acetone for 2 minutes, peroxidase quenched in a 0.1% sodium azide, 0.09% hydrogen peroxide PBS for ten minutes and washed three times in PBS. Sections were incubated with 10% horse serum (PAA Laboratories) for twenty minutes, followed by application of 1:10 dilution of the primary antibody (either CD4 or CD8, clones identified above) in 10% horse serum for 1 hour. Slides were washed 3 times in PBS, followed by application of 2–4 drops of horse anti-mouse IgG horseradish peroxidase secondary antibody (ImmPRESS^™^ HRP Anti-Mouse IgG (Peroxidase) Polymer Detection Kit, Vector Laboratories) and incubated for 30 minutes. Labels were visualized with NovaRed peroxidase (Vector Laboratories). Sections were counterstained in Mayer’s Hematoxylin, dehydrated in ethanol and xylene, and sealed with a coverslip. Non-specific background was evaluated with a duplicate section receiving an isotype control primary antibody (Canine CD3) (negative control). For intestinal sections, the total number of CD4 or CD8 positive leukocytes were counted in 15 randomly selected villi (400um stretch of lamina propria) from all cats.

### *In situ* hybridization (RNAscope)

A novel *in situ* hybridization technique designated as RNAscope (Advanced Cell Diagnostics, Hayward, CA) was used to visualize the presence and location of active viral transcription (viral RNA) in tissues harvested from infected cats. A set of anti-sense FIV specific RNA probes comprised of 120 Z pairs targeting 953–8764 of FIV-C template genome (Genbank AF474246.1) was developed by contractual arrangement with Advanced Cell Diagnostics. Each pair of probes forms a hybridization focus that specifically binds a preamplifier and subsequent amplifiers that can be visualized by chromogenic alkaline phosphatase brown substrate. This assay was performed according to manufacturers protocols with the following specific conditions. Fresh MLN, spleen, and SI tissues from all FIV-positive and one FIV-negative cat were fixed in 10% buffered formalin for 24 hours, embedded in paraffin wax, and sectioned at 4um on positively charged glass slides. Samples were slowly submerged in lightly boiling Pretreatment Two (Advanced Cell Diagnostics) for 15 minutes, followed by application and incubation of Pretreatment Three (Advanced Cell Diagnostics) at 40°C for 30 minutes. A probe specific for a feline housekeeping gene RNA (*Felis catus* peptidylprolyl isomerase B) and for *Bacillus subtilis* strain SMY dihydrodipicolinate reductase (dapB) gene were also generated by Advanced Cell Diagnostics as positive and negative controls respectively. To quantify positive cells in each microanatomical region, the number of chromogenic dots per 400x high power field were counted in 4 high power (400x) fields in the follicular, paracortical, and medullary sinus domains of the MLNs; the follicular and red pulp domains of the spleen; and the follicular and villus lamina proprial regions of the intestine from each cat.

### Statistics

Graphical numerical data is presented as the mean of three or more values with the standard deviation or range represented by error bars. Statistical differences were determined by unpaired Student’s t-tests. A p value < 0.05 was considered to be statistically significant. Statistics were performed with Prism 6 software (GraphPad Software, Inc., La Jolla, CA). In comparisons where less than three data points were available, statistics could not be performed and data are graphically presented as the mean and whiskers representing the range of values. A minimum data set is available as a supplemental file (S1 Dataset).

## Supporting information

S1 Dataset Minimum data set. (XLSX)
